# Patterns of Palliative Radiotherapy Utilization for Patients With Metastatic Breast Cancer in Harare, Zimbabwe

**DOI:** 10.1200/GO.20.00656

**Published:** 2021-08-03

**Authors:** Melinda Mushonga, Anna Mary Nyakabau, Ntokozo Ndlovu, Hari Subramaniam Iyer, Jennifer Ruth Bellon, Caroline Kanda, Sandra Ndarukwa-Jambwa, Fallon Chipidza, Rudo Makunike-Mutasa, David Muchuweti, Edwin G. Muguti, Shekinah Nefreteri Cluff Elmore

**Affiliations:** ^1^ Sally Mugabe Central Hospital, Harare, Zimbabwe; ^2^ Parirenyatwa Hospital Radiotherapy Centre, Harare, Zimbabwe; ^3^ Department of Oncology, Faculty of Medicine and Health Sciences, University of Zimbabwe, Harare, Zimbabwe; ^4^ Cancerserve Trust, Harare, Zimbabwe; ^5^ Harvard T.H. Chan School of Public Health, Boston, MA; ^6^ Dana-Farber Cancer Institute, Boston, MA; ^7^ Harvard Radiation Oncology Program, Boston, MA; ^8^ Department of Pathology, University of Zimbabwe, Faculty of Medicine and Health Sciences, Harare, Zimbabwe; ^9^ Department of Surgery, University of Zimbabwe, Faculty of Medicine and Health Sciences, Harare, Zimbabwe; ^10^ Department of Radiation Oncology, University of North Carolina at Chapel Hill, Chapel Hill, NC

## Abstract

In sub-Saharan Africa, radiotherapy (RT) utilization and delivery patterns have not been extensively studied in patients with metastatic breast cancer.

**METHODS:**

A retrospective cohort study of female patients with metastatic breast cancer seen at Parirenyatwa Radiotherapy Centre in Zimbabwe from 2014 to 2018 was conducted. Demographics, pathology, staging, and treatment data were abstracted through chart review. Fisher's exact test and chi-squared test of independence were used to compare proportions, and independent two-sample *t*-tests were used to compare means.

**RESULTS:**

Of 351 patients with breast cancer, 152 (43%) had metastatic disease, median age 51 years (interquartile range: 43-61 years). Of those with metastatic disease, 30 patients (20%) received radiation to various metastatic sites: 16 spine; three nonspine bone metastases; six whole brain; and five chest wall or supraclavicular. Patients who received radiation were younger (46 *v* 52 years; *P* = .019), but did not differ significantly by performance status than those who did not. The most common dose prescription was 30 Gy in 10 fractions (33%). Five (17%) patients had treatment interruption and two (7%) had treatment noncompletion. Province of origin and clinical tumor stage were significant predictors of RT receipt (*P* = .002; and *P* = .018, respectively).

**CONCLUSION:**

A minority of patients with metastatic breast cancer received RT (20%), and these were likely to be younger, with advanced tumor stage, and resided in provinces where RT is available. Conventional courses were generally prescribed. There is a need to strongly consider palliative RT as an option for patients with metastatic breast cancer and use of hypofractionated courses (e.g. 8 Gy in one fraction) may support this goal.

## INTRODUCTION

Breast cancer is the most frequently diagnosed cancer in the world and the second most common cancer among women in most regions of sub-Saharan Africa (SSA).^1,2^ Most patients present with locally advanced or metastatic disease and an increase in the incidence of breast cancer is expected by 2030 in Africa.^3-8^ Palliative interventions, particularly radiotherapy (RT), are integral for the care of these patients. Few studies have assessed the use of palliative RT in SSA, and limited data suggest that RT may be underutilized.^9-12^

**CONTEXT**

**Key Objective**
Patients with breast cancer in resource-limited settings are likely to present with metastatic disease and may benefit from palliative radiotherapy (RT) for symptom control and improved quality of life. Yet, access to RT in these settings may be limited. This study reports patterns of palliative RT use for patients with metastatic breast cancer in a resource-limited setting in sub-Saharan Africa with public-sector RT access.
**Knowledge Generated**
There was a low utilization of RT for patients with metastatic breast cancer (20%) and conventionally fractionated regimens (eg, 30 Gy in 10 fractions) were most common. Receipt of palliative RT was more common for patients residing close to an RT facility.
**Relevance**
The results will provide preliminary data to guide targeted education, institutional guidelines, and prospective trials to improve palliative RT use for patients with metastatic breast cancer in resource-limited settings.

Multiple factors may lead to the potential underutilization of palliative RT.^9-15^ Among them, lack of RT services and challenges in prognosticating survival. This may result in preference for alternative treatment modalities such as systemic therapy by treating physicians.^16-18^ Radiotherapy utilization (RTU) varies between countries because of differences in case mix of cancer types and stage at presentation. However, it is assumed that RT indications should be higher in low-income versus high-income countries (HIC) as most cancers in low-income countries (LIC) are diagnosed at advanced stages, thus more likely to have indications for curative or palliative RT. Palliative RTU rates, defined as the proportion of patients with metastatic disease who may need RT, are an estimated 47%-56% in HIC.^19,20^

Substantial progress in the availability of RT facilities has been made in some African countries but availability overall has remained low.^12,13,21,22^ There is an expectation for RTU to be high in patients with breast cancer in Zimbabwe because diagnosis is often at advanced stage.^3,7^ Zimbabwe has three linear accelerators available to patients at Parirenyatwa Radiotherapy Centre (RTC) oncology unit and a total of six for approximately 16 million people. There are no data on patterns of RTU and delivery for patients with metastatic breast cancer in Zimbabwe or other SSA countries. This study reports on the proportion of patients with metastatic breast cancer at RTC who received palliative RT, factors associated with receipt of palliative RT, and the patterns of RT delivery. The data can inform evidence-based guidelines for palliative RT delivery for patients with metastatic breast cancer in low-resource settings.

## METHODS

### Study Setting, Population, and Variables

A retrospective cohort study was constructed of female patients with breast cancer who were seen at Parirenyatwa Radiotherapy Centre (RTC) in Harare, Zimbabwe, from January 2014 to December 2018.

Demographics, pathology, staging, and treatment data were abstracted from hospital record paper files for women with pathologically confirmed breast cancer. For this analysis, only patients with metastatic breast cancer were included. The data used for the analysis included age, Karnofsky performance status, health insurance status, registered address by province, HIV status, tumor characteristics, and treatment details. The registered address by province was recorded from the hospital front sheet and represents either (1) the patient's residence or (2) temporary residence during treatment. Tumor characteristics were recorded from pathology reports and clinical examination was recorded in patient files. These included histologic subtype, grade, immunohistochemistry (IHC), tumor (T) stage, and overall clinical stage. Overall, breast cancer subtype was determined from the IHC without ki67 for further differentiation of luminal A. We used the following classifications for breast cancer subtype: luminal A–like (estrogen receptor [ER]+ and progesterone receptor [PR]+[−] and human epidermal growth factor receptor 2 [HER2−]), ki67 not available for all; luminal B (ER+ and PR+[−] and HER2+); triple-negative (ER− and PR− and HER2−); HER2-enriched (ER− and PR− and HER2+); and hormone receptor–positive/unknown HER2 (ER+ and PR+[unknown or −]), following clinical classifications used in Zimbabwe.

Treatment details included receipt of chemotherapy, surgery, endocrine therapy, and RT. Information on anatomic site, treatment length, treatment noncompletion, and treatment interruption were collected. Treatment interruption was defined as failure to complete scheduled RT sessions within the originally prescribed period according to the RT treatment chart. Treatment noncompletion was defined as failure to complete scheduled fractionation according to the RT treatment chart.

### Statistical Analysis

Patient, disease characteristics, and treatment details were summarized with descriptive statistics and accompanying proportions for respective covariate classes. Fisher's exact test and the chi-squared test of independence were used to compare proportions, and the independent two-sample *t*-test was used to compare means between patients with metastatic disease who received RT and those who did not. A *P* value of < .05 was considered statistically significant. SAS version 9.4 (Cary, NC) was used for the analysis. Ethical approval was obtained from all necessary institutions including the Partners Healthcare Institutional Review Board (Boston, MA) and the Medical Research Council of Zimbabwe.

## RESULTS

**FIG 1:**
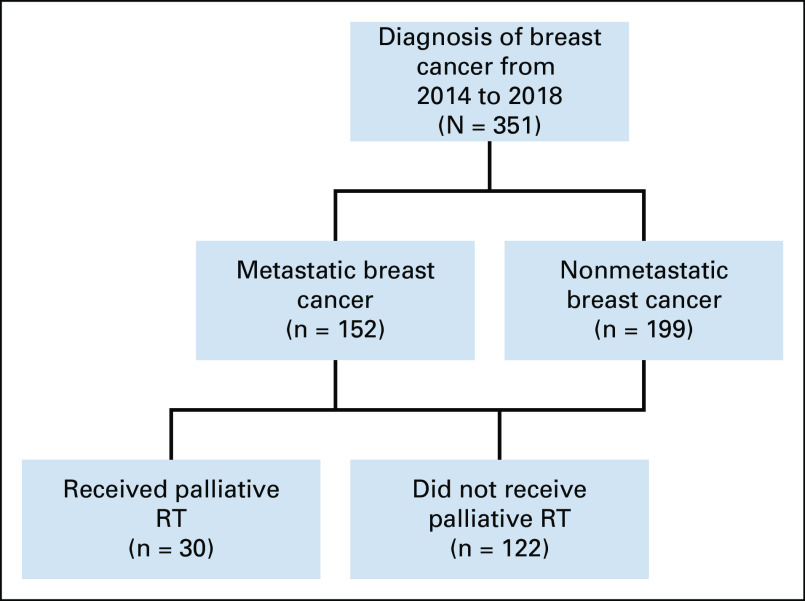
Study flow diagram. RT, radiotherapy.

A total of 351 patients with breast cancer were reviewed for the period defined. 152 (43%) patients had metastatic disease and were included in the analysis as shown in Figure 1. Of those with metastatic disease, 30 patients (19.7%) received radiation.

### Patient and Disease Characteristics

Full demographics are available in Table 1. Patients who received radiation were younger (median: 46 *v* 52 years; *P* = .019), but did not differ significantly by performance status than those who did not. However, performance status was not documented for more than half of the patients (78.51%). Most patients (77%) did not have health insurance. In the full cohort, 17% of patients were known to be HIV-positive. The rates did not differ significantly by receipt of RT (*P* = .66), although nearly one third (30%) did not have documented HIV status. A greater proportion (34%) of patients not receiving RT were registered outside of Harare or Bulawayo, the two RT center locations, compared with those receiving RT (23%, *P* = .002) Ductal subtype and grade I-II histology were the most common for those with the two variables documented. IHC was not available for most patients, 73% in the no RT group and 60% in the RT group; however, those with available results were more likely to be luminal A subtype 21; 14% (*P* = .12). The most common tumor stage in the cohort was T4, although 32.2% of the patients did not have the T size documented. Of those who received RT with a documented and subclassified T4, all eight patients had ulcerated (T4b and T4c) lesions. None of these received palliative RT to the breast. Among the patients with a defined T4 who did not receive RT, 60 (76.0%) had ulcerated lesions (T4b and T4c).

**TABLE 1:**
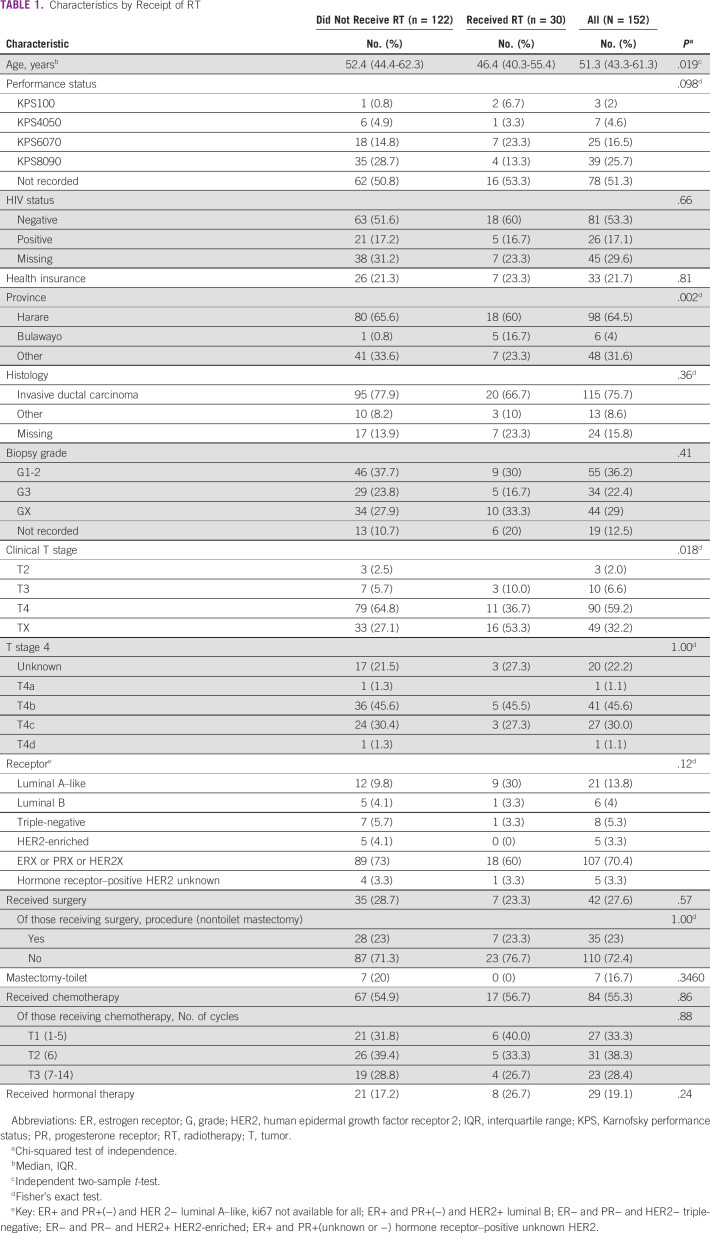
Characteristics by Receipt of RT

### RT

Among patients with metastatic breast cancer, 30 (19.7%) RT courses were prescribed: 16 spine; three nonspine bone metastases; six whole brain; and five chest wall or supraclavicular (Table 2). There were five (17%) who had their RT treatment interrupted and two (7%) patients who did not complete their treatment. Most common treatment courses were 30 Gy in 10 fractions (33.3%); 8 Gy in one fraction (26.7%); and 20 Gy in five fractions (20%).

**TABLE 2:**
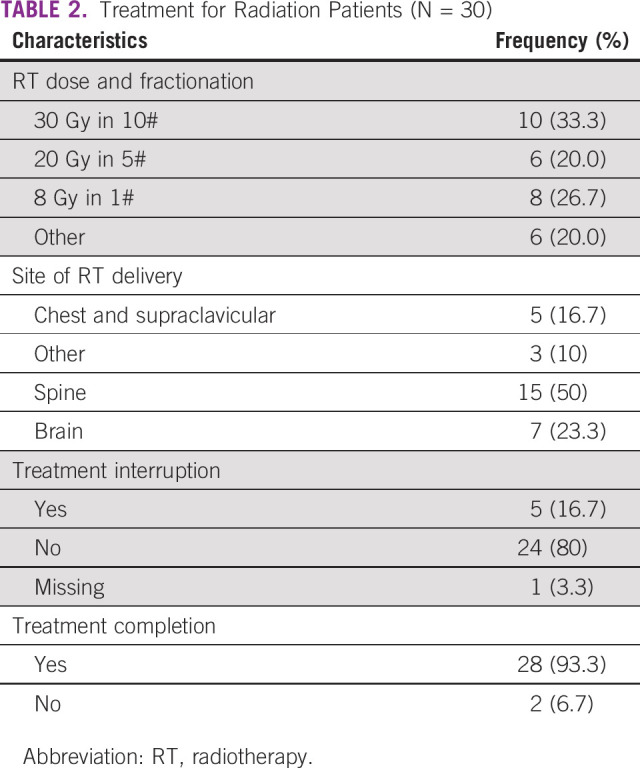
Treatment for Radiation Patients (N = 30)

### Surgery, Chemotherapy, and Endocrine Therapy

Seven (23.3%) patients of the 30 who received palliative RT had breast surgery during the course of their disease. Of these, five later received curative doses of RT to the chest wall and supraclavicular regions. Among patients who did not receive RT, 35/122 (28.7%) had breast surgery. Toilet or palliative mastectomy was documented to have been undertaken in seven patients and the rest did not have the breast surgery type documented (Table 1). Timing of surgery in relationship to other treatments was not consistently documented. The majority of patients in both the RT group and the no RT group received chemotherapy at some time during their treatment course. There were no differences in the number of cycles of chemotherapy among those who received RT versus those who did not receive RT. Endocrine treatment was received by 19% of all patients.

## DISCUSSION

This study describes the patterns of RTU in patients with metastatic breast cancer at a public referral RT center in Zimbabwe. In this study, 150 (43%) patients had metastatic disease a similar finding to previous study from Zimbabwe, which had 41% of patients presenting with stage IV disease.^3^

In patients who had metastatic disease, approximately 20%^30^ received RT. There are no documented studies depicting RTU for patients with metastatic breast cancer in other SSA countries for comparison. In HIC, 30%-50% of the workload in RT departments is attributed to palliative RT.^23,24^ It should follow that the percentage could be larger in LIC since patients with cancer in LIC present commonly with incurable disease, and therefore, symptom relief becomes a priority.^19^ The low utilization of RT may be because of preference of alternative treatment modalities, such as chemotherapy, and thus, additional sensitization of physicians and patients may be important to increase the utilization of palliative RT for patients with metastatic breast cancer.^9,16,18,25^ However, indications for RT were not explicitly defined because of limited documentation and thus it is possible that few patients in the non-RT group had indications for palliative RT. Additionally, RT machine breakdowns may have resulted in treating physicians using alternative treatment options.

A greater proportion (34%) of patients not receiving RT were registered outside of Harare or Bulawayo where RT facilities are available as compared to those receiving RT. The centralization of the RT treatment facilities in Zimbabwe likely results in inaccessibility for many patients who do not live in or have relatives in these areas. Additional RT facilities may help to improve the use of palliative RT, as proposed in a study of patients with breast cancer and palliative RT use where distance from an RT facility was noted as a factor for reduced utilization.^9^ Another likely factor for low RT use is financial barriers experienced by patients, who must pay out of pocket for cancer treatment in Zimbabwe. Baseline investigations are often needed as prior workup before treatments, yet most patients (79% in both the RT and no RT) had no health insurance. In these circumstances, patients may forgo RT because of cost of either preliminary workup or RT costs. Establishing streamlined palliative RT access programs should be considered to ensure prompt, cost-effective access of RT.^12,22,25,26^

The patients who received RT were younger, with a median age of 46 years, as compared to patients who did not receive RT, whose median age was 52 years. Older patients in this setting may be skeptical about RT compared with the younger patients who have a better understanding of RT as a treatment modality. This has been highlighted in similar studies in Zimbabwe where focusing on education regarding the benefits of RT is of paramount importance.^21^

In patients with a defined T stage, T4 was the most common. This is a common phenomenon in LIC where many factors have been reported that result in patients presenting with locally advanced disease as noted by recent studies from Malawi and Haiti.^6,27^ A number of patients had ulcerated lesions, and none received RT to primary site for palliation of expected local symptoms from ulcerated breast lesion. Seven patients received palliative mastectomy in the RT group despite conflicting evidence on the role of surgery in the metastatic setting in patients with breast cancer.^28,29^ It is worthwhile for treating physicians to consider RTU for the breast primary site when feasible to control local symptoms and improve quality of life. Surgical colleagues may be encouraged to view RT as an option. In this setting, multidisciplinary meetings before management may assist in individualized treatment recommendations.

IHC was not available for most patients, potentially confounding associations reported. Luminal A was the most common subtype recorded where immunophenotyping was performed. Luminal A is the least aggressive, is less responsive to chemotherapy, and has a better prognosis.^30,31^ Treatment options include endocrine treatment, with RT as an adjunct for patients with large, fungating, ulcerated breast lesions. Unavailability of IHC may prompt the common use of chemotherapy in comparison to RT as a treatment option in the palliative setting. Another study by our group noted that patients who received both RT and chemotherapy generally received fewer cycles compared with patients who received chemotherapy only.^7^ This could be because of the effectiveness of RT in palliating symptoms that patients did not see the need to continue chemotherapy and do not adhere to further chemotherapy. Some prior data suggested that triple-negative breast cancer was a more common subtype among Black women.^32^ Subsequent studies have suggested triple-negative histology is more common in women with West African ancestry.^32,33^ It is more responsive to chemotherapy and presents in young patients, often with large, ulcerated lesions, a common finding in this cohort. However, most patients in this cohort had luminal subtypes consistent with data from Southern Africa.^6,34-36^ Preference for chemotherapy over RT in some instances may be based on the historical assumption of higher triple-negative prevalence combined with unavailable IHC. It is therefore necessary to continue advocating for access to affordable hormone receptor status testing for all patients with breast cancer. This will guide treatment choices that offer overall clinical utility principally for patients with metastatic breast cancer.

In this study, 20% of patients received RT and the most common site of delivery was the spine. Spine metastases were not elaborated on whether complicated with cord compression or uncomplicated in patient records. Bone metastases are common in breast cancer.^37^ As hypofractionation has become preferred, shorter courses for uncomplicated bone metastasis have become more widely adopted.^38^ This is convenient and cost-effective, yet still underused.^39-41^ In our study, fewer patients had single-fraction treatments, similar to results from a recent Ethiopian study.^42^ Several reasons may justify the choice of longer fractionation. More than 50% of patients who received RT had either brain or metastasis to the spine. The spine metastasis could have been complicated as most patients in the setting present late.^3^ Multifractionated regimens are the standard for patients with brain and complicated spine metastases as opposed to most patients with bone metastasis who benefit from single-fraction treatments.^40,43-46^ Despite longer fractionations, most patients completed the planned RT course. This is encouraging as shorter fractionations increase capacity for additional patients to be treated and reduce RT time, potentially reducing the chances of treatment noncompletion because of machine breakdowns.

The findings are a vital advocacy tool to address identified barriers and challenges to optimal treatment for patients with metastatic breast cancer in low-resource settings. It can potentially guide localized protocol development for countries with similar operational environments. A major limitation is the retrospective nature of the study and frequency of missing data, which is common in SSA, given paper medical record systems.^47^ A prospective cohort study that includes greater documentation of RT indications and patient-reported quality of life is needed.

In conclusion, palliative RT is prompt, inexpensive, and offers effective alleviation of focal symptoms from breast cancer metastases. In addition, hospital attendance can be reduced at the end of life with short treatment regimens, and palliative RT is well tolerated by most patients. Therefore, there is a need to strongly consider RT as an option for more patients with metastatic cancer in Zimbabwe to improve symptom management and overall quality of life, and to expand RT access to more patients in the region.
